# Supreme Court Impacts in Public Health Law: 2024-2025

**DOI:** 10.1017/jme.2025.10138

**Published:** 2025

**Authors:** James G. Hodge

**Affiliations:** Sandra Day O’Connor College of Law, Arizona State University, United States

**Keywords:** Supreme Court, health, public health, rights, impacts

## Abstract

After dispensing major precedents affecting the public’s health in each of its prior three terms, the 2024-2025 term of the US Supreme Court was arguably less impactful amid several unanimous decisions preserving existing jurisprudence (at least in part). However, this is an understatement. While the Court issued key decisions arguably favorable to communal health this prior year it also denied minors access to medical procedures sought by their doctors, diminished diversity, equity, and inclusion (DEI) initiatives in employment, allowed states to deny health providers access to Medicaid because they also provided abortions, disallowed rural hospitals from collecting specific costs for treating low-income patients, and provided a “script” of sorts for executive control of federal health advisory committees.

Amid profound public health challenges emerging in the initial months of President Trump’s second turn in the White House, the US Supreme Court (SCOTUS) took on an array of legal cases presenting opportunities to restore confidence in US constitutional and other laws critical to assuring health outcomes. What the nation needed was a clear, definitive series of decisions from the Court affirming constitutional order amid political chaos. What it got instead was another year of divergent and perplexing opinions obscuring the already cloudy public health legal landscape.

As per Figure 1 and accompanying analyses below, among key decisions this term likely to impact Americans’ health outcomes for years to come SCOTUS (1) denied access to medical treatments prescribed by doctors to minors with gender dysphoria, accentuating the legislature’s role in setting medical standards of care; (2) diminished diversity, equity, and inclusion (DEI) initiatives in employment by rejecting distinctions of majority versus minority discrimination under Title VII; (3) denied access to patients seeking to judicially challenge states’ rejection of specific health service providers that also conduct abortions; and (4) rejected rural hospitals’ pleas for a more favorable reimbursement formula for costs absorbed in treating at risk, low-income Medicare patients.

**Figure 1.**
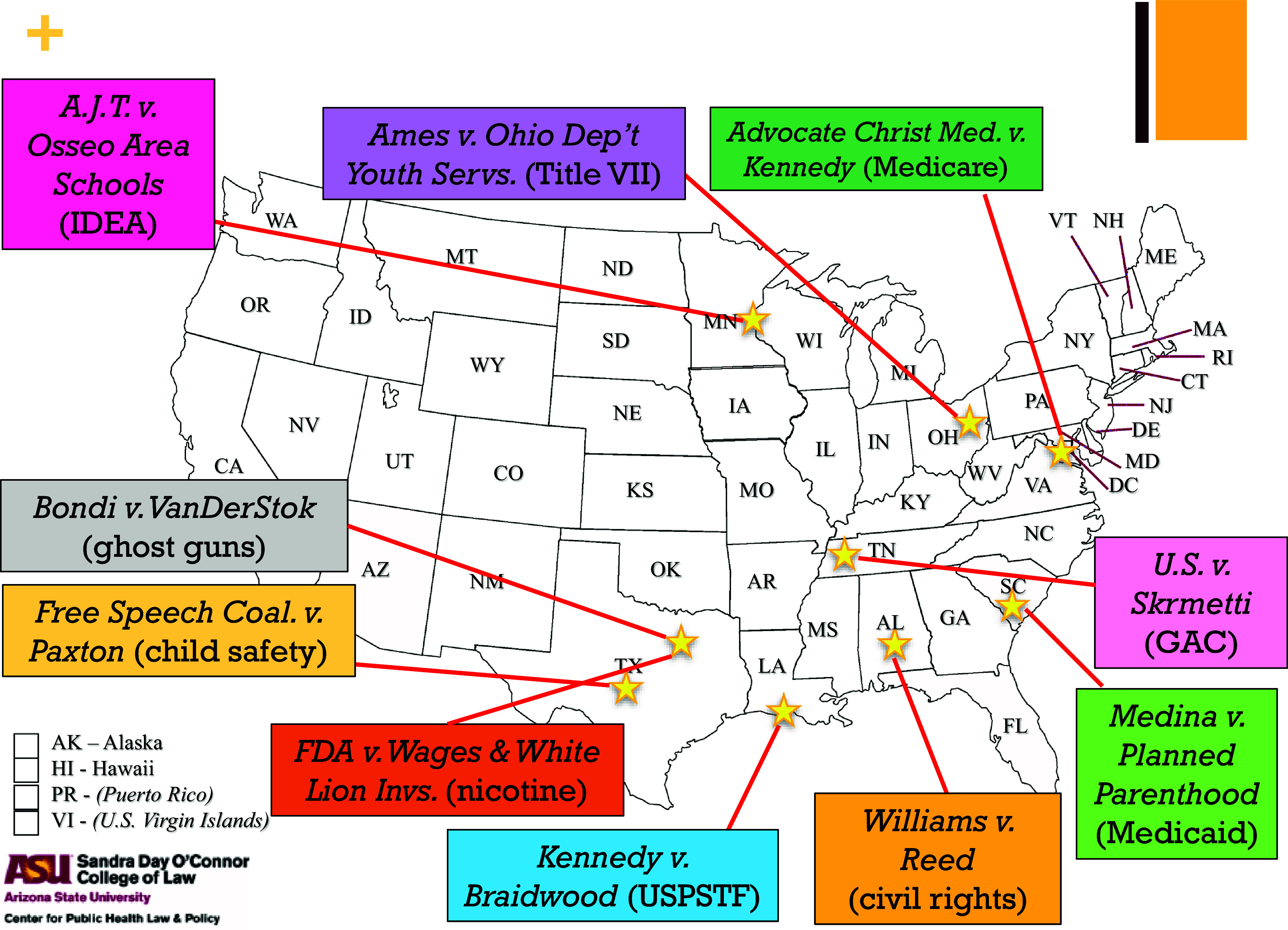
SCOTUS Health Law Cases 2024–2025.

As per prior terms, however, not all of the Court’s decisions were adverse to communal health. In a widely-watched case, the Court affirmed the *status quo* of the influential US Preventive Services Task Force (USPSTF) against a challenge regarding how its members were appointed. Although it was a clear “win” for health care service delivery, the Court’s decision carries collateral damages. SCOTUS also negotiated its way through a free speech morass surrounding access to online pornography with at least a goal of protecting minors. Harkening back to the COVID-19 pandemic, the Court enhanced Americans’ ability to raise federal claims for constitutional violations underlying administrative denials of health or other benefits. It leveled the playing field for disabled kids seeking federal disability protections following successful proofs of insufficient educational plans. And, in two cases with roughly similar outcomes (albeit on different grounds), SCOTUS affirmed the Food and Drug Administration’s (FDA) regulatory prowess to curtail specific vaping products attractive to kids and the capacity of the federal Bureau of Alcohol, Tobacco, Firearms and Explosives (ATF) to regulate “ghost gun” kits as “weapons” under federal statutory law.

**Gender Affirming Care**. In *U.S. v. Skrmetti* ^1^ the Supreme Court determined that Tennessee Senate Bill 1 did not violate equal protection principles in disallowing specific treatments for gender dysphoria among transgender minors. Writing for a 6–3 majority, Chief Justice Roberts determined that the bill met constitutional muster under the Court’s highly permissive “rational basis” test because the legislature drew classifications among patients solely based on “age” and “medical use,” neither of which are “suspect classes,” and not “sex,” which is.^2^ Working around the Court’s 2020 decision in *Bostock v. Clayton County^3^* (holding that employees fired for being gay or transgender violates Congressional anti-discrimination prohibitions based on “sex”^4^), SCOTUS validated Tennessee’s prohibition of access to specific treatments for gender dysphoria, based in part on emerging evidence of potential harms of such treatments among youth.^5^ Never mind documented harms of failing to receive such treatments. Endorsing the legislature’s perceptions, the Court avoided clarifying the scope of sex-based discrimination under equal protection. Consequently, over 20 states with similar measures may now effectively determine medical standards of care for transgender youth, irrespective of doctors’ and parents’ concerns for their patients.

**Majority Group Discrimination**. On June 5, 2025, Justice Jackson led a unanimous Court in rejecting distinctions to prove Title VII employment discrimination claims involving persons of majority in *Ames v. Ohio Department of Youth Services.* ^6^ Ames, a White, straight woman, claimed sex-related discrimination by her state employer when she was passed up for promotion and later demoted by her LGBTQ colleagues. The Sixth Circuit Court of Appeals demanded Ames show additional “background circumstances” to prove discrimination because she was a person of majority.^7^ Justice Jackson rejected this premise. “Our case law [clarifies] that the standard for proving disparate treatment under Title VII does not vary” in cases involving majority plaintiffs.^8^ Justice Thomas, joined in concurrence by Justice Gorsuch, would have gone further to eliminate any “judge-made” laws ascertaining Congress’ requirements under Title VII.^9^ The endgame of *Ames* is beyond refute. Employment discrimination affecting minorities or majorities must be assessed under the same standards. In line with extant political pressures and changing regulations via the Equal Employment Opportunity Commission,^10^ explicit DEI efforts in employment settings that distinguish on the bases of sex, race, or other Congressionally-classified criteria are voided.^11^ Similarly, public health allocations, programs, and benefits based on majority-minority distinctions are at great risk of legal rejection as well.

**Medicaid Coverage**. Can Medicaid-covered patients who are cut off from access to care through specific health providers (that also conduct abortions) challenge state-based denials under federal law? “No,” affirmed Justice Gorsuch for a 6–3 majority in *Medina v. Planned Parenthood South Atlantic.* ^12^ Patients may not sue state agencies for refusing to include specific providers under Medicaid via 42 U.S.C. § 1983. While Congress statutorily obligated states providing Medicaid programs to include any “qualified providers,”^13^ it essentially left it to states to determine exactly who qualified. In *Medina*, the South Carolina Department of Health and Human Services withdrew Planned Parenthood from its Medicaid provider list because it performed abortions in select circumstances contrary to new restrictions enacted by the state legislature. ^14^ Whether South Carolina’s decision constituted affirmative health policy was inconsequential. SCOTUS was solely focused on whether a spending clause provision of Congressional law allowed § 1983 challenges. Typically, such claims must be grounded in explicit constitutional violations or clear rights-based statutory language. For plaintiffs to succeed, Justice Gorsuch clarified, “… they must show, at a minimum, that [the key Medicaid provision] §1396a(a)(23)(A) … ‘clear[ly] and unambiguous[ly]’ gives them individual federal rights.”^15^ Finding no express language to this end, unlike its prior case, *Tavleski*, in 2023,^16^ Justice Gorsuch rejected plaintiffs’ claims. And along with it, SCOTUS negated further challenges to key interventions under Medicaid and other federal health laws, substantially setting back patients’ and providers’ interests.

**Rural Health Care**. In *Advocate Christ Medical Center v. Kennedy*,^17^ the Court took a formulaic approach to resolve a long-standing debate over Congress’ defined calculation of the Medicare disproportionate share payments to hospitals by the Centers for Medicare and Medicaid Services (CMS). Prior courts and administrative tribunals had already disallowed expansive assessments of the “Medicaid” portion of the calculation. SCOTUS merely followed suit. In a 7–2 opinion by Justice Barrett,^18^ with dissents filed by Justice Jackson joined by Justice Sotomayor,^19^ the Court limited the scope of Medicare payments to hospitals serving low-income populations. Only specific patients who actually received SSI payments in a given month while hospitalized could be included for purposes of calculating such payments, and not those who were merely eligible for them. Justice Jackson observed how “systemically undercounting low-income patients [via the] formula might cause many such hospitals to close their doors entirely… .”^20^ Poor American patients seeking care especially in rural areas may soon find fewer, if any, good options.

**Public Health Advisory Committees**. Presented with an amorphous argument that USPSTF members were inappropriately appointed under Article II of the US Constitution, a federal district court in Texas attempted to invalidate the Task Force and its recommendations which are key to coverage of essential services via the Affordable Care Act. Wading through constitutional and legislative history, as well as a litany of semantic details, Justice Kavanaugh clarified on June 27 that USPSTF members were “inferior officers,” under the control of Department of Health and Human Services (HHS) Secretary Robert F. Kennedy, Jr. Justice Kavanaugh’s decision for a 6–3 majority in *Kennedy v. Braidwood Management, Inc.*,^21^was indisputably the right call, preserving the legitimacy of a key advisory health committee. An inapposite conclusion would have potentially invalidated years of committee findings and resulting health coverages.

Yet the Court’s reasoning was simultaneously problematic. While preserving the Task Force, Justice Kavanaugh clarified the extensive supervisory control that Secretary Kennedy has over the committee. Kennedy can appoint, remove, or supplant the members anytime. If he disagrees with the Task Force’s findings, Kennedy can effectively ignore them. In light of the Secretary’s sweeping displacement of all of the members of the Advisory Committee for Immunization Practices earlier in June 2025, the Court all but handed over the keys to America’s health insurance coverage to a leading appointee at HHS who is unafraid to clean house at his own (or the President’s) discretion.

**Access to Online Pornography**. Texas, like multiple other states, has sought to limit minors’ access to damaging online pornographic websites through age-based verification requirements set nearly unanimously by the state legislature. No one reasonably objects to keeping minors away from obscene images and material. The problem, claimed porn site industry representatives and others in *Free Speech Coalition, Inc. v. Paxton*,^22^ is that such restrictions also impair adult First Amendment access to legitimate, lawful material online. Seeking to balance competing interests, Justice Thomas for a 6–3 majority adjudged Texas’ age verification requirements constitutionally sound under the Court’s less stringent “intermediate scrutiny” standard.^23^ If SCOTUS applied strict scrutiny to Texas’ bill, as the industry requested and Justice Kagan advocated, the state’s measures would have unquestionably failed. If Texas got its way, seeking a low bar “rational basis” standard, there is no telling what else it (or other states) would have sought to restrict minors’ access to (*e.g.*, websites providing transgender health information). Under the Court’s preexisting intermediate scrutiny test, ^24^ Texas’ “content-neutral” law survived because it “advances important governmental interests unrelated to the suppression of free speech” (*e.g.*, protection of minors)^25^ and “does not burden substantially more speech than necessary to further those interests,”^26^ notwithstanding Justice Kagan’s strong inferences to the contrary.^27^

**Government Health and Other Benefits**. Persons’ abilities to raise constitutional claims countering government malfeasance in distributing unemployment (or other) benefits was addressed in *Williams v. Reed.* ^28^ Multiple Alabamians denied access to unemployment payments extending from job losses during COVID-19 sued the state labor department. Kafkaesque inefficiencies in administering their applications resulted in alleged due process violations brought by the plaintiffs under the aforementioned § 1983^29^ (see *Medina* case discussion above). The Alabama Supreme Court refused to even consider the claims because plaintiffs did not exhaust their administrative remedies as required by state law.^30^ In SCOTUS’ 5–4 opinion on February 21, 2025, Justice Kavanagh identified a catch-22: persons denied benefits were prevented from litigating because they could not exhaust their remedies through the very same agency that denied their claims.^31^ Congress did not intend for § 1983 actions framed in procedural due process to be overridden by state bureaucracy. Though limited in its scope, *Williams* arguably extends federal legal options for persons whose health or other benefits are rejected without sufficient due process even as *Medina* ^32^ limits them absent explicit legislatively recognized rights.

**Disability Discrimination**. For decades the Eighth Circuit Court of Appeals required schoolchildren and their guardians who successfully laid out a case of discrimination under the Individuals with Disabilities Education Act (IDEA)^33^ to meet a higher standard to subsequently prove a case of discrimination under the Americans with Disabilities Act (ADA)^34^ or federal Rehabilitation Act.^35^ Even after proving that a school system discriminated under IDEA’s requirement to craft individualized education plans for disabled students, parties were required by the Eighth Circuit^36^ to demonstrate schools acted in “bad faith or gross misjudgment” to succeed on separate ADA or Rehab Act complaints.^37^ Thousands of kids were denied disability protections, including A.J.T. and her parents claiming compensatory damages for years of IDEA violations by their Minnesota-based school system. Chief Justice Roberts, writing for a unanimous Court, rejected these judicial limitations.^38^ Invoking Congressional intent underlying the IDEA, SCOTUS equalized the rights of disabled kids and their families to federal disability protections under the same standards as other Americans.

**E-Cigarette Nicotine Controls**. In another 9–0 unanimous decision, Justice Alito navigated a complex series of regulatory decisions by FDA denying hundreds of requests by manufacturers to market new e-cigarette flavors. Under the Family Smoking Prevention and Tobacco Control Act of 2009,^39^ FDA was authorized to prohibit tobacco products (including nicotine delivery devices) that may harm the public’s health. Amid shifting FDA guidance under the Act, e-cigarette providers sought to sell a panoply of candy and fruit flavored products, which could be appealing to children. In *FDA v. Wages and White Lion Investments, LLC*,^40^ SCOTUS rebuked an *en banc* panel of the Fifth Circuit Court of Appeals admonishing FDA for denying industry requests.^41^ Instead, SCOTUS affirmed FDA’s extensive authority to approve the sale and marketing of e-cigarettes,^42^ landing a major victory for public health advocates seeking to protect minors from nicotine addictions via products designed to attract their use.

**“Ghost” Guns**. ATF’s ability to regulate “ghost gun” kits as “weapons” under the Gun Control Act of 1968^43^ was affirmed by the Court on March 25 in *Bondi v. Vanderstok.* ^44^ Writing for a 7–2 majority, Justice Gorsuch clarified Congress’ authority allowing ATF to address rampant sales of these kits, rebuffing a challenge largely by gun manufacturers. They claimed that ATF regulations exceeded Congressional intent in violation of the Administrative Procedure Act.^45^ The Court heard nothing of it. Noting rapid escalation of gun kit purchases among consumers who could, in some cases, assemble non-traceable parts in less than 30 minutes, Justice Gorsuch concluded that Congress clearly intended ATF to regulate these kits as “weapons.”^46^ As a result, “ghost guns” may be serialized and tracked, and buyers subjected to background checks, just the same as with other firearms.^47^ Furthermore, those using such weapons for criminal purposes may be more easily held accountable.
